# Non-Alcoholic Fatty Liver Disease Is Associated with an Increased Incidence of Atrial Fibrillation in Patients with Type 2 Diabetes

**DOI:** 10.1371/journal.pone.0057183

**Published:** 2013-02-22

**Authors:** Giovanni Targher, Filippo Valbusa, Stefano Bonapace, Lorenzo Bertolini, Luciano Zenari, Stefano Rodella, Giacomo Zoppini, William Mantovani, Enrico Barbieri, Christopher D. Byrne

**Affiliations:** 1 Division of Endocrinology, Diabetes and Metabolism, Department of Medicine, University and Azienda Ospedaliera Universitaria Integrata of Verona, Verona, Italy; 2 Division of General Medicine, “Sacro Cuore” Hospital of Negrar, Verona, Italy; 3 Division of Cardiology, “Sacro Cuore” Hospital of Negrar, Verona, Italy; 4 Diabetes Unit, “Sacro Cuore” Hospital of Negrar, Verona, Italy; 5 Division of Radiology, “Sacro Cuore” Hospital of Negrar, Verona, Italy; 6 Section of Hygiene and Preventive, Environmental and Occupational Medicine, Department of Public Health and Community Medicine, University of Verona, Verona, Italy; 7 Department of Prevention, Public Health Trust, Trento, Italy; 8 Nutrition and Metabolism, Faculty of Medicine, University of Southampton, Southampton, United Kingdom; 9 Southampton National Institute for Health Research Biomedical Research Centre, University Hospital Southampton, Southampton, United Kingdom; Scientific Directorate, Bambino Hospital, Italy

## Abstract

**Background:**

The relationship between non-alcoholic fatty liver disease (NAFLD) and atrial fibrillation (AF) in type 2 diabetes is currently unknown. We examined the relationship between NAFLD and risk of incident AF in people with type 2 diabetes.

**Methods and Results:**

We prospectively followed for 10 years a random sample of 400 patients with type 2 diabetes, who were free from AF at baseline. A standard 12-lead electrocardiogram was undertaken annually and a diagnosis of incident AF was confirmed in affected participants by a single cardiologist. At baseline, NAFLD was defined by ultrasonographic detection of hepatic steatosis in the absence of other liver diseases. During the 10 years of follow-up, there were 42 (10.5%) incident AF cases. NAFLD was associated with an increased risk of incident AF (odds ratio [OR] 4.49, 95% CI 1.6–12.9, *p*<0.005). Adjustments for age, sex, hypertension and electrocardiographic features (left ventricular hypertrophy and PR interval) did not attenuate the association between NAFLD and incident AF (adjusted-OR 6.38, 95% CI 1.7–24.2, *p* = 0.005). Further adjustment for variables that were included in the 10-year Framingham Heart Study-derived AF risk score did not appreciably weaken this association. Other independent predictors of AF were older age, longer PR interval and left ventricular hypertrophy.

**Conclusions:**

Our results indicate that ultrasound-diagnosed NAFLD is strongly associated with an increased incidence of AF in patients with type 2 diabetes even after adjustment for important clinical risk factors for AF.

## Introduction

Non-alcoholic fatty liver disease (NAFLD) has reached epidemic proportions and is the most common cause of chronic liver disease in clinical practice [1], [2]. The prevalence of NAFLD has been estimated to be in the 20 to 35% range in the general adult population in Western countries and is almost certainly increasing [1], [2]. Compared with nondiabetic subjects, patients with type 2 diabetes seem to be at increased risk for developing NAFLD and certainly have a higher risk for developing advanced fibrosis and cirrhosis. It has been estimated that approximately 60 to 70% of persons with type 2 diabetes have some form of NAFLD [1]–[3].

To date, growing clinical evidence indicates that NAFLD is linked to an increased risk of cardiovascular disease (CVD) both in patients without diabetes and in those with type 2 diabetes [3], [4]. Recent studies also suggest that NAFLD is associated with early left ventricular (LV) diastolic dysfunction, independently of hypertension and other cardiometabolic risk factors [5]–[7]. More recently, two large community-based cohort studies that used serum levels of gamma-glutamyltransferase (GGT) to diagnose NAFLD have shown that this disease is associated with an increased incidence of heart failure, independently of several established risk factors [8], [9].

In parallel, it is well recognized that atrial fibrillation (AF) is the most common sustained arrhythmia and its prevalence is expected to rise substantially over the next few decades because of ageing population and improvements in cardiovascular treatments [10], [11]. The prevalence of AF increases from about 1% in individuals less than 55 years of age to about 10–12% in those older than 80 years of age [10]. Along with older age, many pathologic conditions such as obesity, hypertension, coronary heart disease, heart failure and valvular heart disease have been reported to be among the strongest risk factors for new-onset AF [12]–[14], which is a disease associated with high rates of hospitalisation and death [10], [15].

Thus, although NAFLD correlates with abnormalities in cardiac structure and function and shares with AF multiple cardiometabolic risk factors, there is currently a lack of available information on the relationship between NAFLD and AF in people with type 2 diabetes, a group of individuals in which these two diseases are highly prevalent. Very recently, the Framingham Heart Study investigators have reported an independent association between mildly elevated serum transaminase concentrations, a surrogate marker of NAFLD, and increased risk of new-onset AF in the community [16].

The aim of this study was to test the hypothesis that NAFLD as diagnosed by ultrasonography (the most widely used imaging test for diagnosing hepatic steatosis) predicts subsequent development of incident AF in people with type 2 diabetes.

## Materials and Methods

### Participants

**Figure 1:**
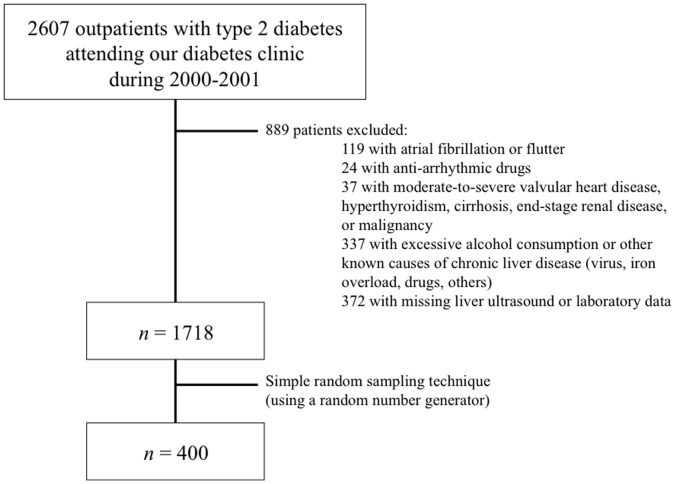
Details of the study design.

In this exploratory analysis, we followed for 10 years a sample of 400 patients with type 2 diabetes, who were clinically free from AF at baseline. As detailed in Figure 1, these participants were selected by a simple random sampling technique (using a random number generator) from the whole cohort (*n* = 1,718) of outpatients with type 2 diabetes attending the diabetes clinic at the ‘Sacro Cuore’ Hospital of Negrar (Verona) during 2000–2001, after excluding subjects who did not meet the inclusion criteria for the study. In particular, we excluded (1) patients who had a history of AF or atrial flutter, (2) those who were taking any anti-arrhythmic drugs, (3) those who had a history of previous moderate-to-severe aortic and mitral valvular disease, hyperthyroidism, malignancy and end-stage renal disease, (4) those who had known causes of chronic liver disease (i.e., alcohol-induced or drug-induced liver disease, viral hepatitis, hemochromatosis or other known causes of liver diseases), and (5) those with missing liver ultrasound or laboratory data.

The sample size of this study was calculated with the specific aim of constructing a confidence interval around the incidence proportion of AF in patients with analogous characteristics. In a similar patient cohort the proportion with AF has been estimated to be approximately 7% [14]. Therefore, with a precision of 2.5% and a confidence interval of 95%, we calculated that a sample size of 400 patients would be needed, taking also into account a cumulative proportion of losses to follow-up of 20%. Thus, a sample size of 400 patients from a population of 1,718 patients produces a 95% confidence interval equal to the population proportion, plus or minus 2.5%, when the estimated proportion of patients with AF is 7% and the expected cumulative proportion of losses to follow-up is 20%. As specified in the Results section (1^st^ paragraph), the random sampling procedure allowed us to select a sample of 400 patients that was well representative of the 1,718 type 2 diabetic patients initially eligible.

All participants were periodically seen at the diabetes clinic (every 6–12 months) for medical examinations of glycemic control, chronic diabetic complications and routine 12-lead electrocardiograms (ECG). The ascertainment at the end of the follow-up period (January 2011) for the whole sample was 100%.

The local ethics committee of the ‘Sacro Cuore’ Hospital of Negrar approved the study and all participants gave their written informed consent for participation in this medical research.

### Clinical and Laboratory Data

BMI was calculated by dividing weight in kilograms by the square of height in meters. Blood pressure was measured in duplicate by a physician with a mercury sphygmomanometer (at the right upper arm using an appropriate cuff size) after patient had been seated quietly for at least 5 minutes. Subjects were considered to have hypertension if their blood pressure was ≥140/90 mmHg or if they were taking any anti-hypertensive drugs. Information on medical history, alcohol consumption, smoking and use of medications was obtained from all patients by interviews during medical examinations.

Venous blood was drawn in the morning after an overnight fast. Serum liver enzymes, lipids and other biochemical blood measurements were determined by standard laboratory procedures (DAX 96; Bayer Diagnostics, Milan, Italy). Most participants (92% of total) had serum liver enzyme levels within the reference ranges in our laboratory. No participants had seropositivity for viral hepatitis B and C. LDL-cholesterol was calculated by the Friedewald’s equation. HbA1c was measured by an automated high-performance liquid chromatography analyzer (HA-8140; Menarini Diagnostics, Florence, Italy); the upper limit of normal for our laboratory was 5.8%. Albuminuria was measured by an immuno-nephelometric method on a morning spot urine sample and expressed as the albumin-to-creatinine ratio.

At baseline, the diagnosis of left ventricular hypertrophy (LVH) was made by a single cardiologist on the basis of a resting 12-lead ECG according to Sokolow-Lyon’s voltage criteria (SV1+RV5 or RV6≥3.5 mV) and/or Cornell’s voltage criteria (SV3+RaVL >2.0 mV in women and >2.8 mV in men, respectively) [17]. In all participants the electrocardiographic PR interval was also recorded. Coronary heart disease (CHD) was defined as a documented history of myocardial infarction, angina, coronary artery bypass grafts, percutaneous trans-luminal coronary angioplasty or typical ECG abnormalities (according to the Minnesota code). The history of previous congestive heart failure and mild valvular heart disease were confirmed by reviewing medical records of the hospital, including diagnostic symptoms patterns, echocardiograms and results of other laboratory exams. Chronic kidney disease (CKD) was defined as the presence of abnormal albuminuria (urine albumin-to-creatinine ratio ≥30 mg/g) and/or glomerular filtration rate <60 ml/min/1.73 m^2^ as estimated by the four-variable Modification of Diet in Renal Disease (MDRD) study equation [18].

### Liver and Carotid Ultrasonography

At baseline, hepatic ultrasonography was performed in all patients by a single experienced radiologist, who was blinded to the participants’ details. Hepatic steatosis was diagnosed on the basis of characteristic sonographic features, i.e., evidence of diffuse hyper-echogenicity of the liver relative to the kidneys, ultrasound beam attenuation and poor visualization of intra-hepatic vessel borders and diaphragm [19]. It is known that ultrasonography has good sensitivity and specificity for detecting moderate and severe hepatic steatosis (∼90–95%), but its sensitivity is reduced when the hepatic fat infiltration upon liver biopsy is <33% [19]. Semi-quantitative sonographic scoring for the degree of hepatic steatosis (mild, moderate or severe) was not available in this study. Grading of hepatic fat content using ultrasonography has been used in previous studies but remains somewhat subjective [19].

The presence of atherosclerotic plaques (i.e., stenosis of 30% or more) at the level of either internal or common carotid arteries was diagnosed by echo-Doppler scanning, which was performed by a single specialist physician, who was blind to subjects’ characteristics.

### Diagnosis of Incident Atrial Fibrillation

At baseline, all participants were free from AF as documented by a standard 12-lead ECG. A 24-hour Holter monitor examination was not routinely performed either at baseline or during the follow-up period. During the follow-up, participants were diagnosed with AF if AF or atrial flutter was present on a standard ECG that was obtained either from a routine clinic examination in our diabetes clinic (i.e., a 12-lead resting ECG was performed annually in all participants) or from reviewing hospital and physician charts from all participants. The diagnosis of AF was confirmed in affected participants by an experienced cardiologist, who was blinded to clinical characteristics of participants, including NAFLD status.

### Statistical Analysis

Data are expressed as means ± SD, medians (interquartile range) or percentages. Skewed variables (serum liver enzymes, triglycerides and diabetes duration) were transformed using natural logarithmic transformation to improve normality prior to analysis. The unpaired-*t* test (for continuous variables) and the chi-squared test or the Fisher’s exact test when appropriate (for categorical variables) were used to analyze the differences among the characteristics of the participants at the time of enrollment in relation to their status of either future development of AF (Table 1) or presence of NAFLD at baseline (Table 2). Binary logistic regression analysis was used to study the association between NAFLD and incident AF (Table 3). We preferred to perform a logistic regression analysis instead of a time-dependent Cox regression analysis since in presence of a small number of events a time-to-event type of analysis, such as Cox regression, is more susceptible to bias than binary logistic regression analysis when adjusted for predictor variables since there is the potential for a marked difference in time to event in the exposed versus the unexposed group. In addition, since the precise time to event (AF) may not be known in some people with asymptomatic AF (e.g. in those with slow AF), we undertook logistic regression analysis. Nevertheless, our results remained essentially unchanged when we used either Cox regression analysis or robust Poisson regression analysis. Compared with logistic regression analysis, both of these time-dependent regression analyses yielded similar estimates of regression coefficients for the association between NAFLD and risk of AF (data not shown). For prediction of incident AF, men and women were combined and first-order interaction terms for sex-by-NAFLD interactions on risk for AF were examined. Because the interactions were not statistically significant (*p* = 0.38), a sex-pooled multivariable logistic regression analysis was used. Four forced-entry regression models were performed: an unadjusted model; a model adjusted for age and sex (model 1); a model further adjusted for hypertension (blood pressure ≥140/90 mmHg or treatment), and electrocardiographic LVH and PR interval (model 2); and, finally, a regression model (model 3) adjusted for variables included in the 10-year Framingham Heart Study-derived AF risk score (i.e. age, sex, BMI, systolic BP, hypertension treatment, electrocardiographic PR interval and history of heart failure) [20]. As sensitivity analyses, we restricted our association analysis between NAFLD and incident AF to patients at the baseline examination who did not have a documented history of ischemic heart disease and heart failure (*n* = 353). A Kaplan-Meier analysis of incidence curves for AF during 10 years of follow-up was undertaken; in patients with, and without NAFLD at baseline. Differences between groups was tested by the log-rank test.

**Table 1:** Baseline clinical characteristics of participants stratified by atrial fibrillation (AF) status at follow-up.

	No AF at follow-up	AF at follow-up	*p* value
Sex (male/female, *n*)	211/147	24/18	0.85
Age (years)	63±9	69±9	<0.001
BMI (kg/m^2^)	29.6±4.7	30.0±5.1	0.54
Diabetes duration (years)	5.0 (1–17)	9.0 (1–24)	<0.01
Systolic BP (mmHg)	139±15	147±15	<0.001
Diastolic BP (mmHg)	81±7	80±8	0.81
Pulse pressure (mmHg)	58±12	67±13	<0.001
Hemoglobin A1c (%)	7.7±1.6	7.7±1.7	0.92
HDL-cholesterol (mmol/L)	1.24±0.3	1.32±0.3	0.16
LDL-cholesterol (mmol/L)	2.84±1.3	2.81±1.3	0.82
Triglycerides (mmol/L)	1.45 (0.41–2.49)	1.41 (0.52–2.42)	0.20
ALT (U/L)	24 (5–39)	27 (8–44)	0.56
GGT (U/L)	29 (6–53)	39 (7–90)	<0.05
PR interval (msec)	166±23	210±36	<0.001
Current smokers (%)	21	17	0.45
History of coronary heart disease (%)	9	10	0.98
History of mild valvular disease (%)	1	2	0.38
History of congestive heart failure (%)	1	10	<0.001
Hypertension (%)	68	90	<0.01
Electrocardiographic LVH (%)	21	52	<0.001
Carotid artery stenoses ≥30% (%)	50	81	<0.005
Chronic kidney disease (%)	24	36	0.10
ACE-inhibitors or sartans (%)	61	71	0.18
Calcium channel blockers (%)	22	31	0.20
Alpha blockers (%)	5	12	0.08
Beta blockers (%)	12	14	0.70
Diuretics (%)	26	41	<0.05
Anti-platelet drugs (%)	62	76	0.28
Lipid-lowering drugs (%)	27	19	0.23
Oral hypoglycemic drugs (%)	71	69	0.67
Insulin therapy (%)	20	26	0.33
NAFLD (%)	68	90	<0.001

Sample size, *n* = 400. Data are means ± SD, medians (interquartile range) or percentages. Differences between the groups were tested by the unpaired-*t* test (for continuous variables), the chi-squared or the Fisher’s exact test (for categorical variables) when appropriate.

ALT, alanine aminotransferase; GGT, gamma-glutamyl-transferase; LVH, left ventricular hypertrophy; NAFLD, non-alcoholic fatty liver disease.

Hypertension was defined as blood pressure ≥140/90 mmHg and/or treatment. Electrocardiographic LVH was diagnosed according to Sokolow-Lyon and/or Cornell’s voltage criteria.

**Table 2:** Baseline clinical characteristics of participants stratified by NAFLD status at baseline.

	WithoutNAFLD	WithNAFLD	*p* value
Sex (male/female, *n*)	68/51	167/114	0.73
Age (years)	64±9	63±9	0.28
BMI (kg/m^2^)	27.1±4.4	30.7±4.5	<0.001
Diabetes duration (years)	7.0 (1−10)	5.0 (1−13)	0.68
Systolic BP (mmHg)	138±14	141±15	<0.05
Diastolic BP (mmHg)	80±7	81±7	0.28
Pulse pressure (mmHg)	57±12	60±13	<0.05
Hemoglobin A1c (%)	7.6±1.6	7.8±1.6	0.42
HDL-cholesterol (mmol/L)	1.30±0.3	1.24±0.3	<0.05
LDL-cholesterol (mmol/L)	2.88±1.3	3.02±1.3	0.43
Triglycerides (mmol/L)	1.26 (0.96−1.81)	1.56 (1.14−2.22)	<0.001
ALT (U/L)	22 (16−31)	30 (24−41)	<0.05
GGT (U/L)	28 (20−43)	33 (25−50)	<0.05
PR interval (msec)	161±25	173±29	<0.01
Current smokers (%)	17	22	0.07
History of coronary heartdisease (%)	9	9	0.95
History of mild valvulardisease (%)	1	1	0.95
History of congestive heartfailure (%)	1	3	0.50
Hypertension (%)	65	73	<0.05
ElectrocardiographicLVH (%)	23	25	0.86
Carotid artery stenoses≥30% (%)	54	55	0.93
Chronic kidney disease (%)	19	23	0.06
ACE-inhibitors or sartans (%)	54	66	<0.05
Calcium channel blockers (%)	27	27	0.98
Alpha blockers (%)	5	7	0.91
Beta blockers (%)	19	12	0.12
Diuretics (%)	33	31	0.79
Anti-platelet drugs (%)	66	61	0.27
Lipid-lowering drugs (%)	27	27	0.97
Oral hypoglycemic drugs (%)	63	74	<0.05
Insulin therapy (%)	22	20	0.48

Sample size, *n* = 400. Data are means ± SD, medians (interquartile range) or percentages.

**Table 3:** Logistic regression models for NAFLD as a predictor for development of AF in patients with type 2 diabetes.

Logistic Regression Models	Odds Ratios (95% CI)	*p* value
**NAFLD (yes ** ***vs*** **. no)**		
unadjusted model	4.49 (1.6–12.9)	<0.005
adjusted model 1	5.40 (1.8–15.9)	<0.005
adjusted model 2	6.38 (1.7–24.2)	= 0.005
adjusted model 3	4.96 (1.4–17.0)	= 0.01
**Other independent predictors of incident AF in regression model 2**		
Age (years)	1.06 (1.01–1.12)	<0.01
Electrocardiographic PR interval (msec)	1.05 (1.03–1.06)	<0.001
Electrocardiographic LVH (yes *vs*. no)	4.29 (1.8–10.4)	<0.001

Sample size, *n* = 400. Data are expressed as odds ratios ±95% confidence intervals as assessed by univariable (unadjusted) or multivariable logistic regression analyses.

Other covariates included in multivariable logistic regression models were as follows: **model 1**: age and sex; **model 2:** age, sex, hypertension (blood pressure ≥140/90 mmHg or treatment), electrocardiographic PR interval and LVH; **model 3:** adjustment for variables included in the 10-year Framingham Heart Study-derived AF risk score (i.e. age, sex, BMI, systolic BP, hypertension treatment, electrocardiographic PR interval and history of heart failure).

All analyses were performed using statistical package SPSS 19.0 and statistical significance was assessed at the two-tailed 0.05 threshold.

## Results

Overall, the 400 randomly selected participants did not significantly differ from the initially eligible sample of 1,718 type 2 diabetic patients in terms of baseline demographics (age: 64±10 *vs*. 66±4 years; male sex: 58.7 *vs*. 60.5%; duration of diabetes: 6±7 *vs*. 8±4 years), HbA1c (7.6±1.6 *vs*. 7.4±1.0%), documented history of CHD (9.3 *vs*. 10.6%) and heart failure (2 *vs*. 3.5%), proportion of obesity (43.9 *vs*. 46.7%), hypertension (70 *vs*. 73.6%) and NAFLD on ultrasound (70.2 *vs*. 72.4%).

Of the 400 participants included in the study, 281 (70.2%) patients met the clinical criteria for diagnosis of NAFLD (i.e., hepatic steatosis on ultrasound among persons who drank less than 20 g/day of alcohol, and who did not have viral hepatitis, drug-induced liver disease, iron overload or other secondary causes of liver disease) and 119 (29.8%) patients did not.

During the 10 years of follow-up, 42 patients developed incident AF (i.e., cumulative incidence of 10.5%). The baseline characteristics of participants stratified by AF status at follow-up are displayed in Table 1. At baseline, patients who developed AF at follow-up were older, had longer duration of diabetes, longer electrocardiographic PR interval, and greater frequencies of hypertension, electrocardiographic LVH and carotid artery stenoses ≥30% than those who did not. Patients who developed AF at follow-up were also more likely to have a documented history of heart failure and had higher values of systolic BP and pulse pressure. Notably, 90% of patients who developed AF at follow-up had NAFLD on ultrasound at baseline. Patients who developed AF also had higher serum GGT levels, although the vast majority of patients (∼90%) had baseline serum ALT and GGT levels within the laboratory reference ranges. Sex, BMI, smoking, serum lipids, HbA1c, CKD, history of previous CHD and mild valvular heart disease, and use of ACE-inhibitors, angiotensin receptor antagonists, beta blockers, lipid-lowering, anti-platelet and hypoglycemic drugs did not significantly differ between the groups.

As expected, when the study participants were grouped according to their NAFLD status at baseline (Table 2), patients with NAFLD were more likely to be obese, to be hypertensive, and had higher systolic BP, higher pulse pressure, higher plasma triglycerides and lower HDL-cholesterol than those without NAFLD. They also were more frequently treated with oral hypoglycemic drugs and ACE-inhibitors or angiotensin receptor antagonists and had higher serum liver enzyme levels, although the vast majority of patients with NAFLD had normal serum ALT and GGT levels.

**Figure 2:**
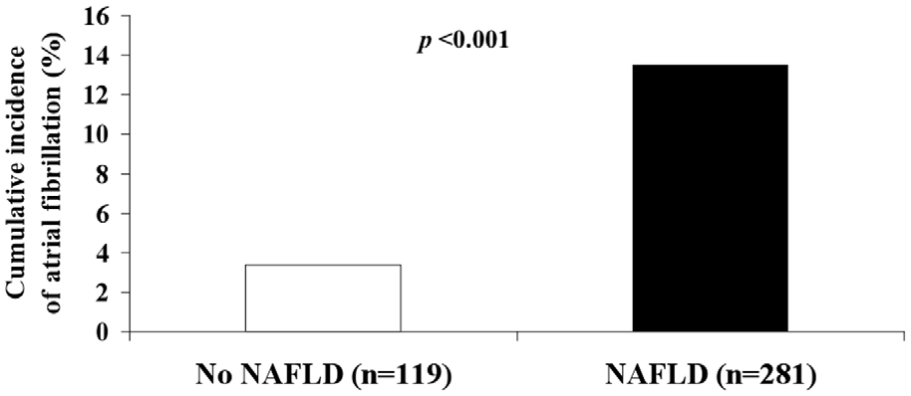
Cumulative incidence rates of atrial fibrillation by NAFLD status.

Notably, as shown in Figure 2, there was also a marked difference in the overall cumulative incidence of AF in patients with NAFLD compared with those without NAFLD (*p*<0.001).

**Figure 3:**
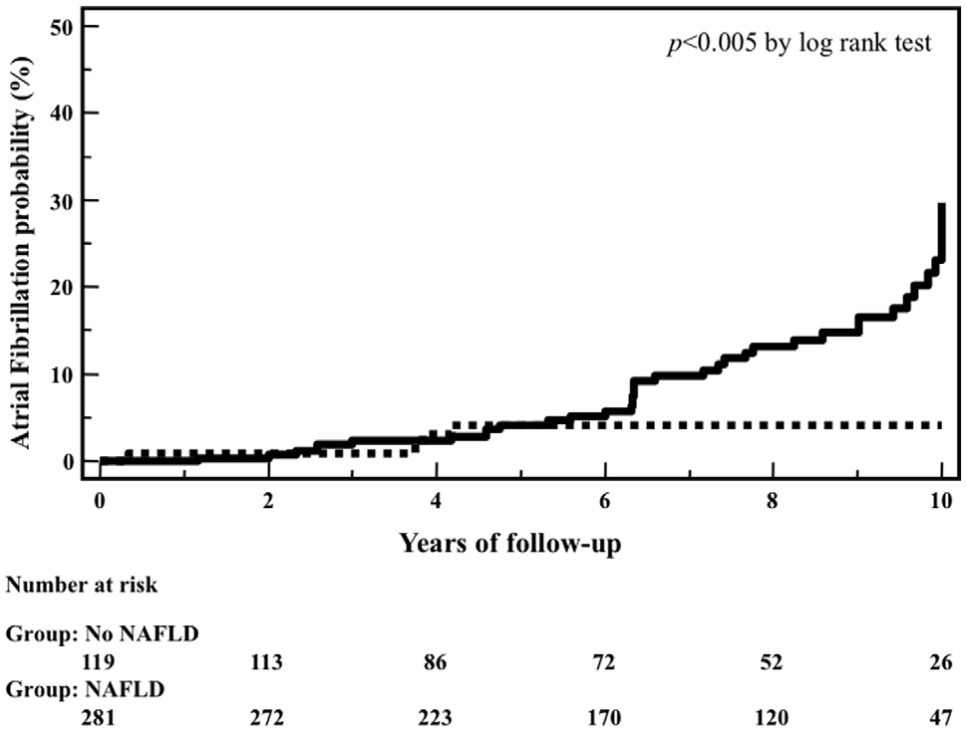
Incidence curves for atrial fibrillation during follow-up, in patients with (solid line) and without (dotted line) NAFLD at baseline.

Figure 3 shows a Kaplan-Meier analysis of incidence curves for AF during 10 years of follow-up in patients with and without NAFLD at baseline. The difference between the two groups was statistically significant and the incidence of AF increased markedly after 6 years of follow-up (*p*<0.005 by the log-rank test).

Table 3 shows the effect of the adjustment for known risk factors on the relationship between NAFLD and risk of incident AF. In univariate analysis (unadjusted model), NAFLD was significantly associated with an increased risk of incident AF. After adjustment for age and sex (model 1), NAFLD maintained a significant association with risk of incident AF. Importantly, the strength of the association between NAFLD and incident AF was not attenuated after additional adjustment for hypertension and electrocardiographic features, i.e. LVH and PR interval (model 2). Notably, in this regression model, other independent predictors of incident AF were older age, LVH and longer PR interval (Table 3). As also shown in Table 3, in a less parsimonious regression model (model 3), the adjustment for variables that were included in the 10-year Framingham Heart Study-derived AF risk score did not appreciably weaken the association between NAFLD and incident AF. However, given the relatively small number of events, the results of this latter regression model should be interpreted with some caution.

Notably, the significant association between NAFLD and increased risk of incident AF remained essentially unchanged even after excluding those (*n* = 47) with documented history of CHD and heart failure: unadjusted model (OR 4.03, 95% CI 1.4–11.6, *p*<0.01), adjusted model 1 (adjusted-OR 4.83, 95% CI 1.6–14.5, *p*<0.01), model 2 (adjusted-OR 4.05, 95% CI 1.1–15.3, *p*<0.05) and model 3 (adjusted-OR 3.78, 95% CI 1.1–13.2, *p*<0.05), respectively.

We also conducted other sensitivity analyses to evaluate the robustness of our findings (*p* values for interaction >0.15 in all subgroups analyses). Almost identical results were found when the results were stratified by sex (OR 2.98, 95% CI 1.1–12.2, for women, and OR 10.4, 95% CI 1.4–80 for men, respectively); by age (OR 8.62, 95% CI 1.1–65 for those aged ≤70 years, and OR 3.94, 95% CI 1.1–14.5 for those older than 70 years of age); by status of electrocardiographic PR interval (OR 3.43, 95% CI 1.1–14.6 for those with PR interval <200 msec, and OR 6.01, 95% CI 1.2–29.7 for those with PR interval ≥200 msec); and by electrocardiographic LVH status (OR 5.31, 95% CI 1.2–25.0 for those without LVH, and OR 4.23, 95% CI 1.02–18.2 for those with LVH, respectively).

## Discussion

NAFLD and AF are two pathologic conditions that are highly prevalent in Western countries and that share multiple cardiometabolic risk factors. Presently, the published research on the association between AF and NAFLD (or liver function tests) is sparse. In a large retrospective cohort study, it has been reported that the prevalence of ALT elevations (i.e. defined as serum ALT >40 U/L), as surrogate markers of NAFLD, among a routine clinical care population with AF was high (i.e. 27.6%), although the incidence of new persistent and significant ALT elevations was uncommon [21]. More interestingly, the Framingham Heart Study investigators have recently shown that moderately elevated serum ALT or AST levels (>40 U/L for either marker) were independently associated with an increased incidence of AF over a 8-year follow-up period in a community-based cohort of 3,744 adults, who were free of clinical heart failure at baseline [16].

To our knowledge, this is the first prospective study to examine the role of NAFLD as detected by ultrasonography (which is a more accurate measure of liver fat than serum transaminase levels) in predicting development of incident AF in patients with type 2 diabetes, who were clinically free from AF at baseline. The major finding of our study was that NAFLD was significantly associated with an increased risk of incident AF during a follow-up period of 10 years. Notably, and more importantly, this association was independent of numerous clinical risk factors for AF.

In accordance with previously published reports, we found that older age, LVH and longer PR interval on ECG (i.e. a measure of left atrial size) were strong predictors of incident AF [12]–[14], [22], [23]. It is well known that LVH causes LV dysfunction and left atrial enlargement, which may lead to fibrosis and electrical remodelling of the atrium, providing a pathophysiological substrate for subsequent development of AF [10], [24]. Recently, the Framingham Heart Study investigators published a clinical risk score for development of AF in 10 years that incorporated the presence of age, sex, BMI, systolic BP, hypertension treatment, longer PR interval and history of heart failure [20]. Similarly, the Atherosclerosis Risk in Communities study showed that a 10-year clinical risk score incorporating age, race, smoking, systolic BP, hypertension treatment, electrocardiographic LVH, electrocardiographic left atrial enlargement, diabetes, CHD and heart failure was predictive for development of AF in a multi-ethnic, community-based cohort of individuals [25].

Although there are few data on cardiac function among patients with NAFLD, preliminary evidence indicates that there is a strong relationship between NAFLD and early LV diastolic dysfunction in both non-diabetic and type 2 diabetic individuals [5]–[7]. It is likely that LV diastolic dysfunction plays a role in AF pathogenesis either by increasing pressure that can affect stretch receptors in pulmonary veins triggers and other areas of the atria or by inducing direct structural changes in atrial myocardium [10], [24]. Interestingly, two large population-based studies have also shown that moderately elevated serum GGT levels, as surrogate markers of NAFLD, are independently associated with an increased risk of incident heart failure [8], [9]. Collectively, as reported above, our findings confirm and extend to patients with type 2 diabetes, using liver ultrasound for diagnosing NAFLD, the recent results reported by Sinner *et al.* [16] demonstrating that NAFLD (as detected by serum transaminase levels) is an independent predictor of new-onset AF in the adult general population.

The underlying mechanisms responsible for the association between NAFLD and increased risk of incident AF require further study. Speculatively, they could include some of the following. Firstly, the association between NAFLD and incident AF is simply a consequence of the shared risk factors and comorbid conditions. However, it is important to underline that in our study NAFLD was associated with an increased risk of incident AF, independently of age, sex, hypertension, electrocardiographic LVH and other clinical risk factors included in the 10-year Framingham Heart Study-derived AF risk score. The odds ratio was not attenuated after adjustment for these potential confounders, thus suggesting that the increased risk of incident AF associated with NAFLD, cannot be fully explained by these shared AF risk factors. Again, the increased risk of AF associated with NAFLD also remained, even after excluding participants with a documented history of previous CHD and heart failure. Secondly, it could be postulated that NAFLD is a marker of ectopic fat accumulation in other tissues, including both the myocardium and pericardium. Rijzewijk *et al*. [26] and Ng *et al*. [27] showed that the intra-myocardial fat content, as detected by proton magnetic resonance spectroscopy, was greater in patients with type 2 diabetes than in nondiabetic controls, and was associated with LV diastolic dysfunction. Interestingly, in the study by Rijzewijk *et al*. [26] there was also a significant, positive association between intra-myocardial and intra-hepatic fat content. Recently, it has been also reported that increased pericardial fat volume was associated with both increased left atrial dimensions [28] and increased prevalence of AF [29], independently of multiple established risk factors. Moreover, Shin *et al*. reported that total and inter-atrial epicardial adipose tissues were larger in AF patients than in matched controls and were independently associated with left atrial remodeling among patients with AF [30]. Preliminary experimental evidence suggests that adipocytes from epicardial or retro-sternal adipose tissues could directly modulate the electrophysiological properties and ion currents, causing higher arrhythmogenesis, in isolated rabbit left atrial myocytes [31]. Thirdly, because in our study NAFLD was associated with increased AF incidence, independently of multiple potential confounders, it is also possible to speculate that NAFLD is not only associated with the risk of AF as the consequence of the shared risk factors but that NAFLD *per se* might partly contribute to the development and persistence of AF. This process might occur through the systemic release of pathogenic mediators from the steatotic and inflamed liver, including C-reactive protein, interleukin-6, tumor necrosis factor-alpha, plasminogen activator inhibitor-1 and other inflammatory cytokines. Importantly, several studies have shown that these pathogenic mediators are remarkably higher in patients with NAFLD than in those without [6], [7], [32], and may play a role in the development and persistence of AF, possibly by inducing structural and/or electrical remodeling of the atria [33]–[36]. These pathways may represent a novel pathogenic mechanism by which structural changes resulting from chronic inflammation can perpetuate AF. These findings require further testing and confirmation in larger clinical trials. Nevertheless, these pathways might provide a potential target for pharmacological interruption or reversal of atrial structural remodeling [33]–[36].

Our study has some important limitations. First, our cohort comprised of type 2 diabetic patients of European extraction, so that the results cannot be generalized directly to other ethnic groups. Second, there were a relatively small number of clinical events during the follow-up and, therefore, the results should be interpreted with some caution. Third, the diagnosis of NAFLD was based on ultrasonography that is relatively insensitive to the presence of smaller amounts of hepatic steatosis (<33% liver fat infiltration) and that cannot distinguish NASH from other forms of NAFLD (although, that said, the overall sensitivity and specificity of ultrasonography for detecting moderate and severe hepatic steatosis are ∼85% and ∼95% respectively, when compared to liver biopsy as a gold-standard) [19]. Although some non-differential misclassification of NAFLD on the basis of ultrasonography is likely (i.e., some of the control patients with diabetes could have mild hepatic steatosis and undetected NAFLD, despite normal serum liver enzymes and a negative ultrasonography examination); this limitation would serve to attenuate the magnitude of our effect measures towards the null. Thus, we reason that our results can probably be considered a conservative estimate of the relationship between NAFLD and increased AF incidence. Since hepatic ultrasonography was assessed at baseline only, we could not investigate the relationship of changes (development or resolution) in hepatic steatosis over time to incident AF risk. Fourth, the diagnosis of LVH was based on widely accepted ECG criteria (that have a very high specificity but a relatively low sensitivity when compared with echocardiographic findings) [17]. Unfortunately, no echocardiographic measurements were available in this study. However, our data have been also adjusted for systolic BP and hypertension treatment, which are likely to capture almost all patients with LVH not detected by classical ECG voltage criteria. In addition, it is important to recognise that the additional incorporation of echocardiographic measurements only slightly improved the predictive ability of the 10-year Framingham Heart Study-derived risk score for the development of AF [20]. Finally, we cannot exclude residual confounding.

Notwithstanding these limitations, our study has important strengths, including its prospective design, the long duration of follow-up (10 years), the relatively large number of participants of both sexes, the diagnosis of hepatic steatosis by ultrasonography (which was performed in all patients by a single experienced radiologist), the complete nature of the dataset, and the ability to adjust for baseline AF risk factors included in the 10-year Framingham risk prediction model [20].

In conclusion, our study is the first to demonstrate that ultrasound-diagnosed NAFLD is closely associated with an increased incidence of AF in patients with type 2 diabetes, independently of important clinical risk factors for AF. Further studies are needed to confirm this finding in other populations, to elucidate the responsible mechanisms for this association, and to explore whether pharmacological interventions aimed at improving NAFLD effectively reduce the incidence of AF in patients with type 2 diabetes. In the interim, from the perspective of clinical practice, it is important that specialists and practicing clinicians be aware of the link between NAFLD and AF, especially because of the high and growing prevalence of these two pathologies.
